# New and emerging therapies in type 1 diabetes mellitus

**DOI:** 10.1172/JCI205520

**Published:** 2026-05-15

**Authors:** Kevan C. Herold, Carmella Evans-Molina

**Affiliations:** 1 Departments of Immunobiology and Internal Medicine, Yale University, New Haven, Connecticut, USA.; 2 Department of Pediatrics and; 3 Center for Diabetes and Metabolic Diseases, Indiana University School of Medicine, Indianapolis, Indiana, USA.; 4 Roudebush VA Medical Center, Indianapolis, Indiana, USA.

## Abstract

Type 1 diabetes mellitus (T1D) has been recognized as a chronic autoimmune disease for five decades, but therapy has relied on the exogenous replacement of insulin, which is an imperfect substitute for normal β cell function. In recent years, there has been progress in the development of new therapeutics that target the primary causes of the disease: failed immunologic tolerance and β cell killing. One agent, teplizumab, was shown to attenuate loss of β cell function that occurs over time and delay progression to clinical disease in individuals at risk, leading to its regulatory approval in 2022. Other immunologic agents show promise in modulating the immunologic imbalance. Moreover, a role for β cells in T1D pathogenesis has been identified and may be targeted. Now that the first disease-modifying therapeutic agent is available, future studies may involve combinations of agents to extend immunologic tolerance and protect and restore β cells so that lasting metabolic remission can be achieved.

## Introduction

Since the discovery of insulin more than a century ago, the treatment of type 1 diabetes mellitus (T1D) has relied on insulin replacement to compensate for the loss of β cells from autoimmune killing. The application of insulin transformed T1D from a uniformly fatal disease into a medically managed chronic condition, but insulin therapy does not restore physiologic glucose regulation (1). Despite major improvements in insulin pharmacokinetics, algorithms for automated insulin delivery, and continuous glucose monitoring, fewer than one quarter of individuals with T1D achieve the recommended glycemic targets (2). Many remain exposed to the cumulative risks of chronic dysglycemia, resulting in the development of microvascular and macrovascular complications and reduced life expectancy (3). In addition, the complete dependence on exogenous insulin, with nonphysiologic pharmacodynamics, can lead to episodes of hypoglycemia that may be severe and even life threatening. These persistent limitations highlight the need for therapeutic strategies that prevent disease onset or modify its course rather than solely replacing insulin.

Recognition of T1D as an immune-mediated disease fundamentally reshaped the field by enabling risk prediction, disease staging, and immune-directed intervention. Genetic risk can be defined with genetic risk scores that evaluate up to 67 SNPs (4). Longitudinal natural history studies tracking the development of clinical T1D in individuals with high genetic risk established a presymptomatic disease continuum, marked by the development of islet autoimmunity — identified with autoantibodies — and progressive metabolic dysfunction (5, 6).

In this Review, we examine advances in our understanding of the mechanisms driving early and progressive stages of T1D and discuss how insights into disease pathogenesis have led to clinical studies for treatment and prevention. We further explore how this science may identify new strategies to enhance and extend the success that has occurred over the past five decades.

## Disease staging and the metabolic phenotype of T1D

The first discovered signs of autoimmunity in T1D were antibodies that react with islet cells in pancreatic sections and subsequently human islets in suspension (7, 8). Autoantibodies serve as the most reliable biomarkers of immune activation but are not a direct cause of the pathology (9). In contrast, T1D is understood to be a disease of T cell–mediated β cell destruction, where autoreactive T cells infiltrate the islets of Langerhans and directly mediate β cell killing prior to the onset of hyperglycemia. T cells that recognize islet autoantigens such as GAD65, ZnT8, IGRP, and proinsulin can be identified in the peripheral blood from individuals with T1D as well as healthy individuals. In individuals with a high risk for T1D, these cells have a memory phenotype, expand, and home to the pancreas (10, 11). More recent studies have identified T cell exhaustion phenotypes that distinguish individuals with T1D from healthy controls and individuals with slow progression to T1D (12). Among peripheral blood CD8^+^ T cells, the T cell exhaustion signature involves high expression of coinhibitory receptors (e.g., programmed cell death 1 [PD-1]) and low expression of CD127. Islet antigen-specific CD8^+^ T cells show increased expression of eomesodermin (EOMES) and inhibitory receptors, consistent with an exhaustion-like phenotype in individuals with slower versus more rapid disease progression (12, 13). In other analyses, autoreactive T cells have a transcriptional profile and phenotype of stem-like cells that includes expression of T cell factor 1 (Tcf1) but not CD39, and a “multipotency index” based on DNA methylation patterns (14). It is postulated that these cells can be activated to become terminal effector CD8^+^ T cells that kill β cells directly by cytolytic mechanisms or indirectly through production of inflammatory mediators (15). Importantly, these stem-like cells, found in low frequency, can replicate and represent a source of new pathogenic cells.

**Figure 1:**
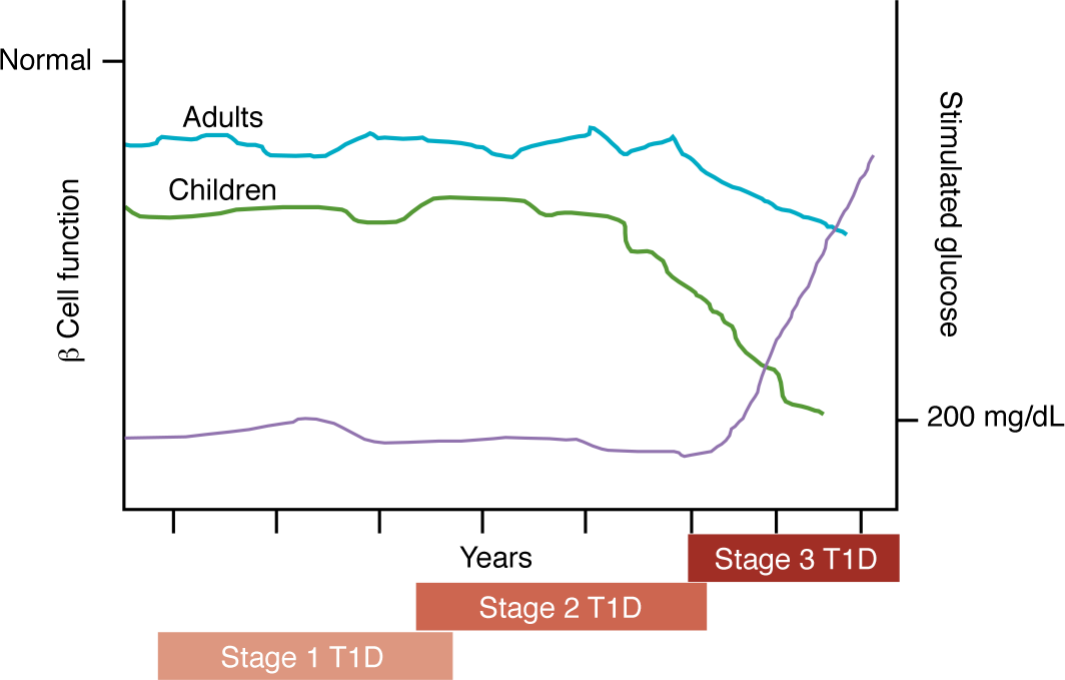
Progression of β cell loss in T1D. The progression from the first discovery of autoantibodies to clinical disease has been described in stages that are defined by metabolic criteria, but these measures are not fixed, and the kinetics of progression are variable among individuals. Even five years before clinical diagnosis, or at the time of stage 1 T1D, β cell function is reduced in response to an oral glucose tolerance test compared with autoantibody-negative individuals without T1D. β Cell function, as assessed by C-peptide responses to an oral glucose tolerance test, shows the greatest decline just prior to the onset of clinical hyperglycemia (stage 3 T1D) (148, 149). Among individuals diagnosed with stage 2 T1D, the median time to stage 3 T1D is approximately two years (119). Notably, younger individuals typically progress more rapidly than do older individuals through early stages to clinical T1D and lose C-peptide more rapidly after stage 3 onset.

On the basis of these and observational studies involving relatives of individuals with T1D (5), the process from initiation to clinical disease has been defined as having three stages. In stage 1 T1D, two or more autoantibodies are present, indicating that the pathologic process has been initiated, but provoked glucose tolerance is clinically normal. However, even at this stage, at least five years before clinical diagnosis, there is impairment of β cell function (Figure 1). Stage 2 T1D is defined as when impaired glucose tolerance occurs with an oral glucose tolerance test. Stage 3 T1D is characterized by metabolic decompensation and fulfills the American Diabetes Association’s glucose thresholds for clinical diagnosis (6). These staging criteria have proven useful for clinical trials focused on early interventions (16, 17). However, they do not identify the kinetics of disease progression, which may differ greatly between individuals, since some people may progress rapidly from stage 1 to clinical diagnosis without a finding of glucose intolerance (stage 2 T1D).

To date, the best determinant of disease progression at each stage of the disease is age: younger individuals typically progress more rapidly than do older individuals through early stages to clinical T1D and lose C-peptide more rapidly after stage 3 onset (Figure 1) (18–22). Curiously, the composition of islet infiltrates also differs by age, and younger individuals have a greater proportion of B lymphocytes within insulitic lesions (23). Autoantibody profiles further contribute to within-stage variability of risk. The presence of autoantibodies against tyrosine phosphatase-related islet antigen 2 (IA2A) identifies individuals who progress more rapidly compared with those without them (24, 25), but the types of autoantibodies are also different among age groups. Younger individuals are most likely to have autoantibodies against insulin, whereas in adolescence and beyond, anti- glutamic acid decarboxylase (GAD) antibodies are the first to appear and are associated with a lower-risk phenotype (24, 26). In addition, HLA genotypes are determinants of autoantibody responses: anti-insulin antibodies are most common in HLA-DR4^+^ individuals, whereas anti-GAD65 antibodies are seen in HLA-DR3^+^ individuals (27, 28). These observations highlight the need for randomized study groups to evaluate targeted interventions.

## Preventatively targeting β cells

Given the inaccessibility of β cells, many insights into the mechanisms of T1D have been derived from studies involving human islets and pancreata from organ donors with T1D and autoantibodies, β cell lines, and murine models of the disease. An important caveat of murine studies is that significant architectural and functional differences exist between human and mouse islets and between affected individuals and models of T1D (29). In NOD mice, the most widely used preclinical model for studies of spontaneous autoimmune diabetes, insulitis is robust, with a dominance of CD4^+^ T cells. In contrast, insulitis in donated T1D human organs, which have been made available by programs like the Network for Pancreatic Organ Donors with Diabetes and the Human Islet Research Network, is modest and dominated by CD8^+^ T cells, but variable between islets in the same pancreas, with variable contributions from other immune cell populations. β Cell loss is not uniform across the pancreas. At diagnosis, insulin-containing islets are found, particularly in individuals diagnosed later in life, and the extent of β cell loss may not correlate with metabolic severity (30, 31). In addition to classical islets, recent analyses of human pancreata show preferential loss of small insulin-containing “endocrine objects” that represent most of the pancreatic endocrine area in early life (32). The observation that clinical onset of T1D does not mark the end of β cell function, but is rather a transition point along a continuum of β cell decline, forms a core justification for the use of disease-modifying therapies aimed at preserving C-peptide. Indeed, the persistence of C-peptide in long-duration T1D is linked with reduced glycemic variability, improved glucose control, and a reduced risk of hypoglycemia and diabetes-associated complications (33–36).

Single-cell studies enabling integrated analysis of the transcriptome, epigenome, proteome, and cellular phenotypes of tissues from human organ donors support a model of tissue–immune cell interactions in which β cell–intrinsic stress within the endocrine pancreas and maladaptive immune responses evolve in parallel and reinforce one another over time (37–39). β Cells themselves can enhance the inflammatory environment. They and other islet cells release chemokines (e.g., CXCL10, CCR9) and cytokines (TNF-α, IFN-γ, IL-6, IL-1β) that recruit and license inflammatory cells. These inflammatory cytokines can also enhance the presentation of antigens, leading to the generation and presentation of neoantigens. Hybrid peptides, in which C-peptide is joined with other β cellular proteins, represent one example of a neoantigen (40, 41). In addition, posttranslational modifications of glucokinase and other β cell constituents are recognized by autoantibodies and T cells from patients (42, 43). Several cellular stress pathways in the β cell are thought to contribute to this feed-forward cycle. Owing to their exceptionally high secretory burden, β cells are particularly vulnerable to disruptions in ER function (44, 45). Perturbations in protein synthesis, folding, and degradation impair insulin secretion and β cell survival and expand the pool of peptides available for immune recognition. In addition, senescent β cells can release proinflammatory cytokines that are chemoattractant and create novel antigenic peptides. Preclinical studies suggest senolytics or senomorphics that can prevent the senescence-associated secretory phenotype (SASP) may have efficacy in preventing disease in NOD mice (46). Since these neoantigens are formed through activation of cell-intrinsic stress pathways and are not expressed in the thymus or bone marrow, central T and B cell tolerance to them is not acquired during immune cell development.

Antigen presentation by islet cells is also enhanced. There is increased expression of HLA class I and II molecules in insulin-containing islets from patients, which is closely linked with proinflammatory cytokine signaling in β cells (47–49).

Collectively, these observations describe a feed-forward cycle in which metabolic stressors may lead to the creation and presentation of neoantigens, and the inflammatory mediators they produce recruit immune cells to the site. In this scenario, immune cells are responders to the products of distressed β cells, rather than initiators, but the immune cells cause further β cell dysfunction, cellular stress, and even killing, which increase metabolic demand on.

**Figure 2:**
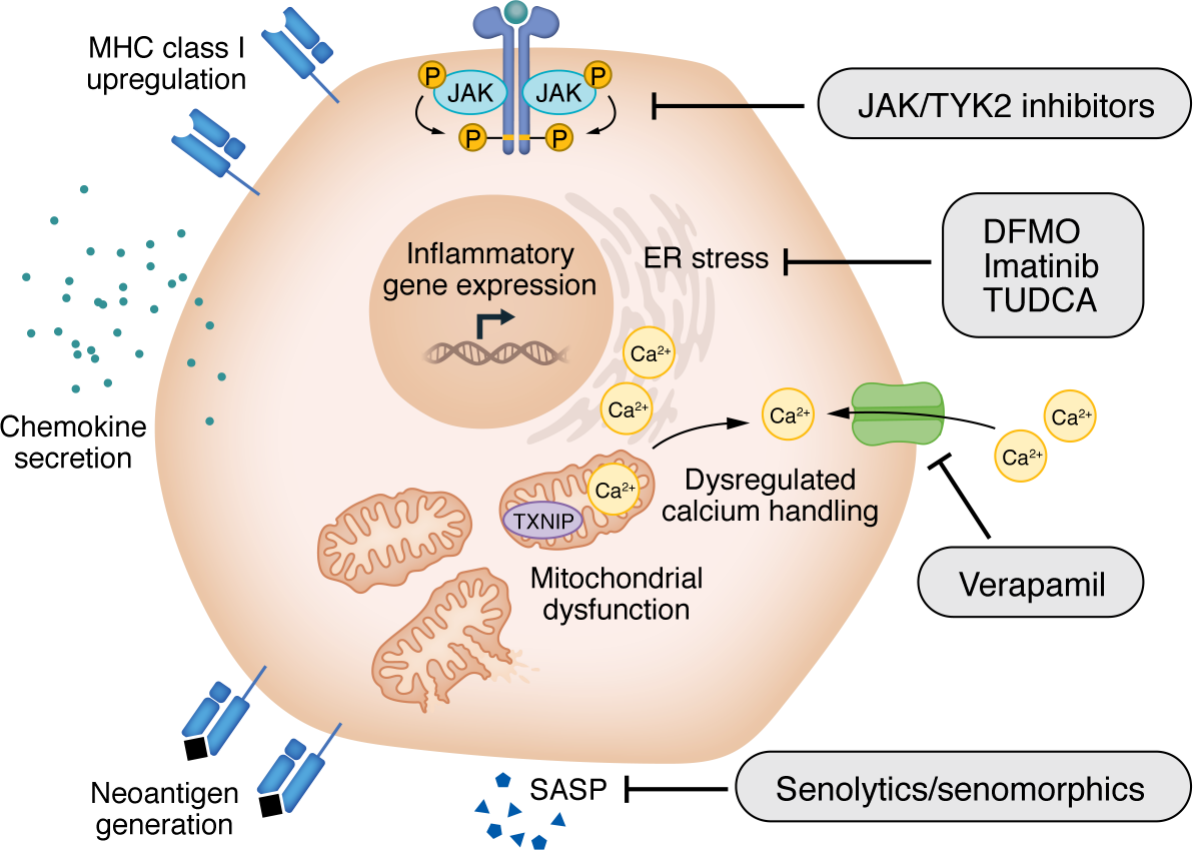
β Cell stress pathways and therapeutic targets in T1D. β Cell–intrinsic stress responses, including ER stress, mitochondrial dysfunction, dysregulated calcium handling, and cellular senescence, converge to activate inflammatory and stress-responsive gene programs and promote neoantigen generation, upregulation of MHC class I molecules, and secretion of chemokines and cytokines that enhance immune recognition and recruitment. Stress-induced signaling further reinforces activated immune responses, creating a feed-forward cycle of β cell dysfunction and immunogenicity. Multiple therapeutic strategies target distinct nodes within this network, including modulators of ER stress (TUDCA, imatinib, DFMO); verapamil, which targets calcium signaling, mitochondrial dysfunction, and TXNIP activation; and senolytics/senomorphics, which target senescence-associated pathways, including SASPs. JAK/TYK2 inhibitors modulate β cell stress and immune activation, highlighting a unique opportunity for interventions that jointly preserve β cell function and dampen immune responses.

Efforts to modulate β cell stress have been explored in preclinical models, and a few notable examples have translated into human clinical trials (Figure 2). Taurourso-deoxycholic acid (TUDCA) is a bile acid and chemical chaperone that reduces β cell ER stress and diabetes development in NOD mice (45). A clinical trial of TUDCA for the treatment of individuals with recent-onset stage 3 T1D has been completed, but the results of this study have not been reported (NCT02218619). Inositol-requiring transmembrane kinase/endoribonuclease 1α (IRE1α) is a bifunctional kinase/endoribonuclease that promotes adaptive unfolded protein response signaling under manageable ER stress but shifts toward proapoptotic signaling when stress is sustained or excessive. Imatinib is an FDA-approved abelson murine leukemia viral oncogene homolog (Abl) kinase inhibitor that was shown to reduce β cell ER stress and apoptosis in preclinical models by antagonizing cellular-Abl–IRE1α interactions, thereby limiting IRE1α hyperactivation (50). A clinical trial showed that 26 weeks of imatinib treatment improved C-peptide in adults with recent-onset stage 3 T1D (51) during drug administration, but the effect was not sustained out to 24 months, suggesting that ongoing treatment may be required (51). In addition, 71% of drug-treated participants had a grade-2 severity or worse adverse event compared with 59% of 22 participants who received placebo. Efforts to identify more specific modulators of IRE1α activity for use in T1D and other inflammatory diseases are underway (52).

Verapamil is an L-type calcium channel blocker shown in preclinical models to reduce β cell intracellular calcium overload (53). Verapamil blocks the expression of thioredoxin-interacting protein (TXNIP), thus preventing TXNIP-mediated inhibition of the antioxidant thioredoxin, which drives NLRP3 inflammasome activation and oxidative stress–associated apoptosis (54, 55). Verapamil has been tested in three clinical trials, with modest yet positive effects on C-peptide in two of the trials. Positive effects were seen in a small trial involving adults with recent-onset stage 3 T1D and in the Hybrid Closed Loop Therapy and Verapamil for Beta Cell Preservation in New-Onset Type 1 Diabetes (CLVer) study that enrolled 88 children and adolescents (aged 8–17 years) with recent-onset T1D (56, 57). However, early analysis from the VERA-T1D study performed within the INNODIA Network failed to show a significant benefit of the drug in 136 adults with recent-onset T1D (58). Other clinical parameters were not improved in the CLVer or VERA-T1D trials. Across these studies, verapamil has been generally well tolerated, suggesting the feasibility of combining the drug with immunomodulatory agents to exert dual-immune and β cell effects. Interestingly, recent preclinical data described effects of verapamil on the immune system, suggesting a potentially complex mechanism of action (54). In addition, the lack of a circulating pharmacodynamic signal of TXNIP inhibition has made it challenging to attribute the beneficial effects of verapamil solely to a change in β cell health and/or stress. Nonetheless, WAVE-T1D is a multicenter clinical trial to be initiated in 2026 that will test the anti–T cell agent antithymocyte globulin (ATG) in combination with either verapamil or the TNF-α inhibitor adalimumab (NCT07061574).

Polyamine biosynthesis is linked with ER stress and inflammatory signaling in β cells (59). Difluoromethylornithine (DFMO) is an irreversible inhibitor of ornithine decarboxylase and the rate-limiting enzyme in polyamine synthesis that is FDA approved for high-risk neuroblastoma and African sleeping sickness. In preclinical models, DFMO reduces β cell ER stress and delays disease onset when administered early, and genetic deletion of ornithine decarboxylase is protective against chemically induced T1D (60, 61). Building on these findings, DFMO, which has an established safety profile in humans, was tested in a dose-ascending study and found to be safe, with positive effects on C-peptide at higher doses (60). Currently, a fully powered phase II trial is being performed in adults and children with recent-onset stage 3 T1D (NCT05594563).

Glucagon-like peptide 1 receptor agonists (GLP-1RAs) have been evaluated in T1D as adjunctive therapies to lower hemoglobin A1c (HbA1c) because of their ability to increase insulin secretion in individuals with residual β cell function. Furthermore, because obesity is increasingly common in people with T1D, there has been interest in the adjunctive use of GLP-1RAs for their effects on weight and satiety. Indeed, clinical trials have shown positive effects on weight, insulin use, HbA1c, and glycemic variability in established T1D (62, 63). Three studies showed benefits of liraglutide or exenatide for patients with established T1D who had residual β cell function but increased rates of hypo-glycemia (64–66).

It is unclear whether GLP-1RAs modulate β cell stress and immunogenicity. Notably, a randomized clinical trial combining IL-21 pathway blockade with liraglutide demonstrated preservation of C-peptide in adults with recent-onset T1D with combination therapy (67). At the end of active treatment, the beneficial effects of combination therapy were greater than those observed with IL-21 or liraglutide alone, suggesting synergy. However, after drug withdrawal, the effects on C-peptide waned. Interestingly, the long-term effects on C-peptide were worse with liraglutide alone compared with the placebo, raising the possibility that driving insulin secretion in the peridiagnostic period could be associated with enhanced immunogenicity or even β cell exhaustion, a possibility that needs to be tested in future studies. In this regard, the “accelerator hypothesis” suggests that obesity could drive the development of islet autoimmunity and T1D in some at-risk populations (68, 69), and, therefore, agents such as GLP-1RAs, which can improve obesity, warrant further investigation in this setting.

## Targeting the autoimmune response

**Figure 3:**
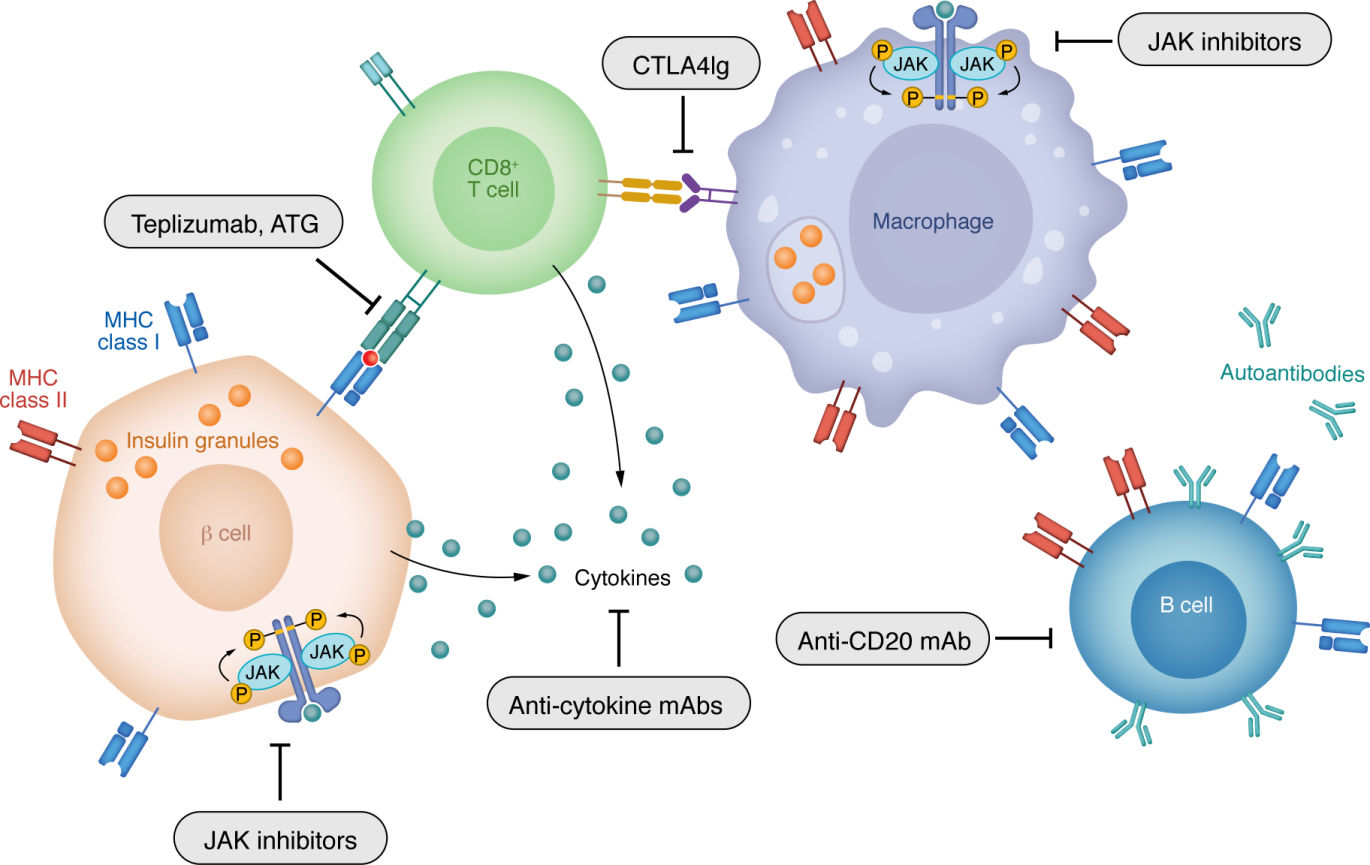
Successful T1D clinical trials targeting immune responses. Therapies directed toward T and B cells as well as inflammatory pathways have been able to improve β cell function and reduce insulin requirements when given at the time of diagnosis of stage 3 T1D. Among the T cell–directed drugs are teplizumab and ATG. CTLA4Ig prevents the activation of T cells. Anti-CD20 mAb rituximab depletes B cells which may present antigens to T cells. JAK inhibitors prevent signaling by inflammatory cytokines and may affect antigen-presenting cells (APCs) as well as β cells themselves.

Macrophages are the first inflammatory cells to infiltrate the islets (70–72). They are in an inflammatory state and sense microbial products in the blood, but the exact mechanisms that lead to their activation are not known (70). In NOD mouse islets, it has been postulated that macrophages present the contents of crinophagosomes — vesicles formed by fusing lysosomes with insulin dense-core granules (DCGs) — thereby creating a tissue-intrinsic mechanism that transforms insulin into an autoantigen (73). In addition, macrophages produce inflammatory cytokines including IL-1β, TNF-α, IL-6, IFN-α, and IFN-γ and lymphotoxin, which have direct damaging effects on β cells. Clinical studies have not directly targeted innate immune cells, but trials aimed at neutralizing their secreted products have been successful (Figure 3). For example, in a randomized, placebo-controlled phase II trial of 84 individuals at stage 3 T1D onset, treatment with golimumab, a fully human anti–TNF-α mAb, for one year improved C-peptide responses and reduced insulin requirements (74). Etanercept, a TNF-α inhibitor, had similar success in a small (*n =* 18) randomized, placebo-controlled trial (75). However, trials of other drugs targeting single inflammatory cytokines, including tocilizumab (anti–IL-6r), canakinumab (anti–IL-1β), or anakinra (IL-1RA) (76, 77), have not been successful, suggesting that there are specific roles for each inflammatory mediator.

In contrast to neutralizing single cytokines, preventing the action of multiple type I and II cytokines can be achieved with JAK inhibitors. There are four members of the JAK family: JAK1, -2, and -3 and tyrosine kinase 2 (TYK2) (78). A feature of JAK inhibitors is the tunable nature of their selective inhibition of cytokine signaling. The beneficial effects of JAK1 and -2 inhibitors in T1D are hypothesized to largely occur through the suppression of cytokines like IFN-α and IFN-γ, while JAK3 has recently also been implicated in type II IFN signaling (79).

In a randomized, placebo-controlled phase II trial (*n =* 91) of baricitinib, an inhibitor of JAK1 and JAK2, C-peptide responses were improved and insulin requirements reduced in individuals with new-onset disease (80). Importantly, baricitinib was well tolerated in this population, despite known risks of cardiovascular events in older individuals with rheumatoid arthritis (81). A second new-onset trial testing the JAK1 inhibitor abrocitinib and the JAK3/Tec kinase inhibitor ritlecitinib is ongoing in TrialNet (NCT05743244), with results anticipated in late 2027, and trials of baricitinib in patients with new-onset disease as well as patients with prediabetes have been initiated (NCT07222332 and NCT07222137). TYK2 signals through IFN-α, IL-23, and IL-12. Its inhibition may theoretically have a more proximal effect on adaptive T cell differentiation, and, notably, genetic polymorphisms in *TYK2* are associated with protection against T1D (82). IFN-α signaling in the β cell increases proinflammatory gene expression, ER stress, and enhances MHC expression (83), suggesting that JAK and TYK2 inhibitors may interrupt the feed-forward cycle of inflammation and dysfunction that exists between the endocrine and immune compartments. Along these lines, several preclinical studies have shown efficacy of TYK2 and JAK inhibitors in reducing β cell immunogenicity (82–85).

To activate T cells, a second, costimulatory signal is needed (86). CTLA4Ig (abatacept) binds to the costimulatory signals CD80 and CD86 and was shown to attenuate loss of C-peptide when given continuously over two years — and even for one year after drug discontinuation (87, 88). However, when given for one year to individuals with stage 1 T1D, the drug’s efficacy was less robust and not sustained (17). In both trials, treatment reduced the frequency of activated T follicular helper (Tfh) cells, and in the latter, CTLA4Ig treatment appeared to inhibit the maturation of memory CD8^+^ and CD4^+^ T cells (89). The differences in clinical outcomes may be related to the specifics of duration of drug treatment or participant clinical characteristics, but they also raise a question about the precise timing of the initial inflammatory responses that lead to disease.

Infiltrating macrophages provide “signal 3,” which affects the differentiation of adaptive immune cells. P40 is a component of IL-12 and IL-23. The former is involved in the differentiation of IFN-γ–producing T cells and the latter with the differentiation of IL-17A–producing cells (90, 91). When the anti-p40 mAb ustekinumab was administered to individuals with new-onset T1D, C-peptide responses improved one year after treatment. Preserved C-peptide responses correlated with reductions in Th cells cosecreting IL-17A and IFN-γ (i.e., Th17.1 cells) and a reduction in a subset of those cells coexpressing IL-2 and granulocyte-macrophage CSF, which are presumed markers of pathogenicity (92).

## First approval of a therapy targeting adaptive immunity

While effector T cells alone can transfer disease in NOD mice, B cells have also been shown to be required for disease development, most likely as autoantigen-presenting cells (93). In a randomized controlled trial, the anti-CD20 mAb rituximab, which depletes B lymphocytes, attenuated the decline in C-peptide for one year after treatment (94, 95). In this trial, B cell depletion was associated with reduced primary responses to the test neoantigen phiX174. However, with B cell recovery, improved C-peptide responses waned and primary antigen responses returned, coinciding with increased frequency of CD4^+^ cells, highlighting the role of B cells in antigen presentation for T cell activation (96, 97). Intervening on this response with sequential combination treatment is being tested in a phase II TrialNet stage 3 study comparing a course of rituximab alone versus ritixumab followed by abatacept in children and adults (NCT03929601).

Initial trials with immune therapies that target T cell function, such as cyclosporine A, azathioprine, and prednisone, showed some efficacy in modulating disease progression, but the toxic side effects in various tissues and cells were unacceptable (98–101). Furthermore, these agents did not induce immune tolerance and required continuous administration, often with waning efficacy. A more lasting approach was needed to avoid chronic immune suppression.

Severe cytokine release due to crosslinking of T cell receptors (TCRs) is associated with anti–T cell mAbs such as OKT3 or high-dose ATG and precludes consideration of these agents for the treatment of T1D. In response, researchers described amino acid modifications of the heavy chain of humanized OKT3 (102–106) that changed the activating properties of the mAb. One of these, hOKT3γ1(Ala-Ala) (teplizumab), markedly reduced the production of TNF-α and IFN-α (102).

**Figure 4:**
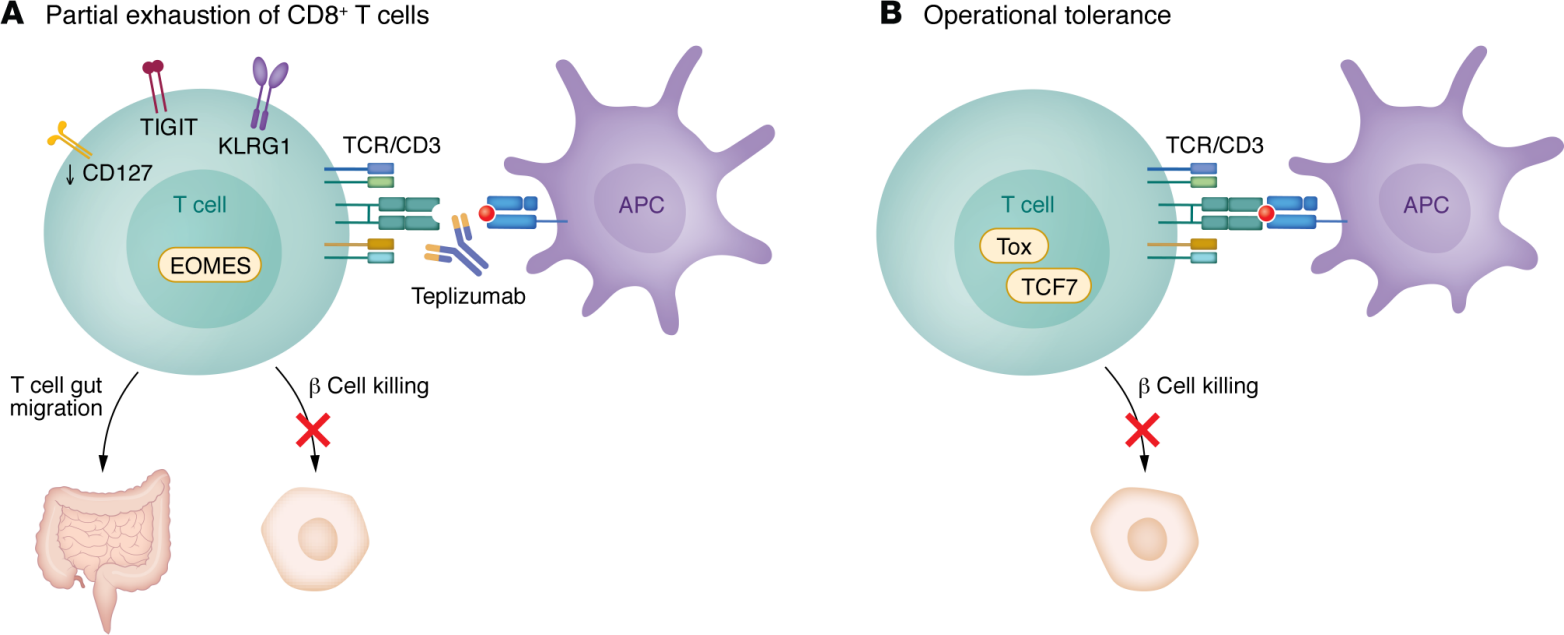
Mechanisms of action of teplizumab. (**A**) The bivalent anti-CD3ε mAb teplizumab delivers a partial agonist signal to T cells. The effects that have been associated with clinical responses have been most pronounced in CD8^+^ T cells, but CD4^+^ T cells are affected as well. Within three months of treatment, there is induction of EOMES, KLRG1, and TIGIT on some CD8^+^ T cells which produce reduced levels of IFN-γ and TNF-α when activated ex vivo (150). In addition, there is reduced expression of CD127, which may account for diminished expansion of autoantigen-specific CD8^+^ T cells (11). (**B**) In the islets of NOD mice treated with teplizumab, immune cells that are found in insulitis disappear but then return to the islets and do not cause β cell killing. These cells express increased levels of TCF1 and Thymocyte Selection Associated High Mobility Group Box (TOX), suggesting a hybrid phenotype with features of stem and exhausted cells (112). These characteristics may account for the long-term responses, designated as “operational tolerance,” that can be seen in some patients treated with teplizumab.

In 1992 and 1994, the first preclinical studies of FcR nonbinding anti-CD3 mAb (mAb 145-2C11) were performed in mice (107). In NOD mice, a five-day course of the mAb durably reversed hyperglycemia (108), suggesting immune tolerance had been achieved. Findings from these preclinical studies had relevance to clinical application: first, the timing of treatment was critical. The mAb treatment was effective only when there were signs of active autoimmunity either during progression of diabetes in NOD mice (>8 weeks of age) or when autologous islets were transplanted (109). Second, the effects of the antibody were not fully accounted for by cell depletion (Figure 4). After infiltrating cells left the islets, they reinfiltrated but did not kill the remaining β cells. This suggested immune tolerance because when immune cells reinfiltrated, the drug was no longer detectable on the cell surface. Studies utilizing NOD mice expressing human CD3 suggested that treatment-induced regulatory CD4^+^ T cells mediated their inhibition of immune responses by TGF-β production (110). CD4^+^ T cells were enriched in the pancreatic lymph nodes of treated mice, which may account for their absence in the peripheral blood of treated patients. Early studies with murine cells showed that the FcR nonbinding mAb triggered the TCR complex but resulted in functional anergy of T cells (111). More recent single-cell studies in NOD mice with remission showed that the T cells that infiltrated islets after treatment exhibited features of naive or stem cells (high expression of *Tcf7* and *Sell*), and others of exhaustion (increased expression of *Tox*) (112). However, the tolerance that is achieved is tenuous and can be rapidly reversed with a single treatment with a checkpoint inhibitor (112, 113).

In clinical studies, teplizumab was given as a 12- or 14-day regimen without ongoing administration. In a phase Ib/IIa trial, participants aged eight years and above with new-onset T1D and treated with a single 12- or 14-day course had improved retention of C-peptide responses and reduced insulin requirements for two years after treatment compared with untreated controls (114, 115). Subsequently, the efficacy of repeating the course of treatment was tested in a trial sponsored by the Immune Tolerance Network, and significant improvement in C-peptide responses and insulin requirements were again seen two years after diagnosis (116). Thus, repeated treatment was safe, but the trial did not formally compare a single versus repeated drug course. In a phase III trial, a shorter treatment course and reduced dosing regimen were compared with previously used dosing regimens in individuals with new-onset stage 3 T1D who were treated at enrollment and again at six months (117, 118). Once again, the full dosing showed improved C-peptide responses at one and two years, but reducing the dose eliminated its efficacy. However, in spite of the improved metabolic responses, the primary outcome of that trial — a composite of HbA1c levels and insulin use — was not met.

As these trials were being conducted, individuals at high risk for clinical T1D, i.e., with stage 2 T1D, who were relatives of patients, were being identified by the NIH National Institute of Diabetes and Digestive and Kidney Diseases (NIDDK) TrialNet. With the understanding that they had a 50% risk of progression to clinical study to determine whether a single 14-day course of treatment with teplizumab would delay the diagnosis of clinical T1D. The trial enrolled 76 participants and randomized them to a single course of teplizumab or placebo. In the drug-treated individuals, the median time to diagnosis with clinical T1D was delayed by 24–48 months at the initial analysis and up to 60 months when the data were analyzed one year later (11, 16, 119). Some treated participants had a very prolonged (i.e., >5 years) delay in disease progression, which has been termed “operational tolerance,” since it represents an arrest of disease progression without the need for continuous treatment (11).

Adverse event profiles with teplizumab treatment have been similar across studies and include transient lymphopenia, rash, and mild cytokine release in some patients (16, 120). Importantly, an infectious disease signal is lacking.

Unlike other anti–T cell mAbs that deplete their targets, depletion alone does not appear to be the primary mechanism accounting for clinical efficacy or duration of teplizumab’s effect. The reduced expansion of autoreactive CD8^+^ T cells that was found in clinical responders in the prevention trial responders may be due to reduced transcription and expression of the IL-7 receptor on CD8^+^ T cells (11). In addition, there was evidence that the activation signal from the drug caused “partial exhaustion” of T cells, characterized by the expression of T cell immunoreceptor with Ig and ITIM domains (TIGIT) and killer cell lectin-like receptor G1 (KLRG1) on EOMES^+^CD8^+^ T cells (119).

On the basis of the efficacy and safety data from five clinical trials, the FDA approved teplizumab in November 2022 to delay clinical T1D in high-risk individuals. This was a significant development for the field, since there had not previously been a therapy available for disease modification. After that initial approval, a phase III trial was completed by Sanofi that enrolled 217 patients 8–18 years of age with stage 3 T1D, who were randomized to drug or placebo treatment for two courses (121). This trial confirmed the effects of drug treatment on preserving β cell function 1.5 years after enrollment, with reduced insulin requirements and similar glycemic control compared with placebo treatment. In November 2025, the European Medicines Agency also approved teplizumab for the delay of stage 3 T1D in high-risk individuals, as did regulatory authorities in the United Kingdom, China, Canada, Israel, Saudi Arabia, United Arab Emirates, and Kuwait.

ATG, another anti–T cell agent, has shown efficacy in changing the course in those with new-onset T1D. ATG showed efficacy in attenuating loss of β cell function at stage 3 onset in adults and children and in those with established disease (122–124). At high doses, the drug induces cytokine release and serum sickness. These events can be mitigated with glucocorticoids, and a more recent trial showed that reduced dosing, with lower rates of adverse events, may be equally efficacious (125). Drug-treated responders exhibited a transient rise in IL-6, IP-10, and TNF-α two weeks after treatment but a durable CD4^+^ exhaustion phenotype (PD-1^+^KLRG1^+^CD57^–^ on all CD4^+^ cells) (126).

## Precision medicine to identify those most likely to respond to treatments

Stages of T1D serve as markers of disease progression in an individual, but there is considerable heterogeneity in the course of disease and even in responses to specific immune therapies. Identifying the features of responders may enable more precise alignment of therapies to individual patients. For example, disease progression along with responses are greater in younger people (i.e., age <15 years) than in older people, suggesting that these therapies would be best tested and utilized in young patients (95, 117, 127).

Baseline parameters may identify optimal treatments. For example, abatacept might be a less optimal consideration for patients with an “immunophenotype” with elevated B cell or neutrophil signatures, and patients with an elevated neutrophil signature showed reduced responses to teplizumab (128, 129). Likewise, Edner et al. found that elevated levels of activated Tfh cells (i.e., ICOS^+^) identified poor responses to treatment with abatacept (130). Microbiomes may also be a basis for selecting treatments, as Xie et al. suggested that immune responses (IgG2) to commensal organisms may help predict time to T1D disease onset and responses to teplizumab (131). This is not surprising, since teplizumab was shown to induce the migration of lymphocytes to the gut wall, and antibiotic treatment can interfere with drug activity (132, 133). Genotypes and the presence of autoantibodies may also be utilized: for example, HLA-DR3 predicts the response to antigen-specific therapy with alum GAD65 (134), and HLA-DR4 predicts the response to teplizumab (16), but the latter has not been confirmed across clinical studies (121). Finally, latent viruses may also affect responses to biologics and, potentially, the choices of therapies. In two separate clinical trials of teplizumab, individuals who were EBV seropositive had better clinical responses to treatment than did participants who were EBV seronegative at enrollment (135). EBV seropositive individuals showed differences in several subsets of T cells at baseline and after teplizumab (135), consistent with features described previously by Faustman (136).

## Future directions

Even successful therapies have not led to a permanent reversal of disease. Cumulative clinical trial experience indicates that a number of strategies may be successful in changing the disease course. Therefore, combinations of agents, together if synergistic (67) but more likely in sequence to avoid immune suppression and with complementary or sustaining mechanisms, are a logical next approach. The choices may be based on the mechanisms of action of the individual agents and the basis for loss of efficacy. An example of this is the TrialNet study combining rituximab followed by CTLA4Ig, which will determine whether preventing T cell activation following B cell depletion can improve outcomes (NCT03929601). Sequential blocking of inflammatory cytokines such as TNF-α and IL-17 or their signaling with a JAK inhibitor may be an effective way to prevent the reemergence of pathologic cells and limit damage to β cells after an initial T or B cell–directed treatment at the time of diagnosis, particularly since the agents are specific in their action and there is clinical experience with long-term use.

Most tested therapies have eliminated, inhibited, or neutralized arms of the immune system. Enhancing immune regulation offers an alternative, with the avoidance of consequences of depletion or inhibitory strategies. Augmentation of Treg function or adoptive cellular therapy with Tregs is supported by findings such as the association of polymorphisms in IL-2RA, which is needed for Treg survival (137), with T1D risk (138), and demonstration of dysfunctional Tregs in patients with T1D (137). However, trials of exogenous IL-2 at varying doses have, to date, failed to modify disease progression (139), which may be attributed to the narrow dosing window that must be achieved to avoid expansion of NK cells, eosinophils, and cytolytic T cells. In addition, expanding patients’ Tregs in vitro with IL-2 can restore the function of these cells, but when the cells were transferred back into the host, clinical efficacy was not achieved (140–142).

Antigen-reactive Tregs may be a more potent and specific solution to this problem, and preclinical studies suggest efficacy (143). Because of their presumed safety, their administration might be considered for prevention or even as part of combination therapy to maintain tolerance, and a trial of proinsulin-specific Tregs has commenced (NCT06919354).

For individuals diagnosed with stage 3 T1D, β cell replacement strategies aimed at restoring lost insulin-producing cells remain under active investigation but face substantial challenges. Allogeneic islet transplantation is limited by immune rejection and low rates of sustained insulin independence, although even partial graft function can reduce the risk of severe hypoglycemia (35, 144). Recent efforts to genetically engineer cadaveric islets to evade immune recognition, including deletion of class I and II MHC molecules and expression of CD47, highlight innovative approaches to overcoming these barriers (145). Parallel advances in stem cell–derived β cell replacement offer a potentially unlimited cell source that can be genetically modified to resist immune-mediated destruction. However, long-term survival and protection from recurrent autoimmunity remain outstanding issues, suggesting that durable metabolic restoration will likely require combination strategies integrating cell replacement with targeted immune therapies (145–147).

Finally, incorporation of immune therapies into endocrine practice represents a new frontier. Care providers need to be trained on and familiar and comfortable with the benefits, risks, and management needed to administer these agents safely. Facilities are needed where they can be safely administered, similar to those now routinely used for immune therapies in other clinical settings. Teplizumab has been approved for individuals at risk, but screening is necessary to identify those to whom the drug can be administered. The optimal setting for screening is with primary care practices rather than endocrine practices. However, with the approval of a treatment at the time of stage 3 diagnosis, this limitation would be resolved, and many more people would have access to disease-modifying therapies.

In summary, great strides have been made in treating the underlying cause of T1D. Individuals who will progress to clinical disease can be identified before the onset of disease, and a drug that can delay this event has received approval. There is greater clarity on the dynamics of the autoimmune response, including the role of β cells as enhancers of the pathology. In the coming years, building on these initial steps may enable more robust efficacy and restoration of a normal metabolic state.

## Conflict of interest

KCH has consulted for Sanofi and Vertex and serves on an advisory board for Sanofi. He is a coinventor on US patent 2022/0041720 and provisional patent 2024/061043. CEM serves on advisory boards for Isla Technologies, DiogenyX, Neurodon and Sanofi and is a coinventor on US patent 6/291,668 and provisional patent 63/285,765.

## Funding support

- NIH grants R01 DK057846, R01 DK 129523, and UG3 DK142189 (to KCH).
- Breakthrough T1D grant 2-SRA-2022-1197-S-B (to KCH).
- NIH grants R01 DK093954 and UC4DK104166 (to CEM).
- VA Merit Award I01BX001733 (to CEM).
- Breakthrough T1D grant 3-SRA-2026-1860-S-B (to CEM).
- Ball Brothers Foundation (to CEM).
- George and Frances Ball Foundation (to CEM).

## Version 1 05/15/2026

Electronic publication
